# Prevalence of Low Back Pain among Undergraduate Physiotherapy Students in Nigeria

**DOI:** 10.1155/2016/1230384

**Published:** 2016-02-03

**Authors:** Grace O. Vincent-Onabajo, Ejiofor Nweze, Fatima Kachalla Gujba, Mamman Ali Masta, Mohammad Usman Ali, Ali Alhaji Modu, Chuka Umeonwuka

**Affiliations:** Department of Medical Rehabilitation (Physiotherapy), College of Medical Sciences, University of Maiduguri, Maiduguri 600001, Borno State, Nigeria

## Abstract

*Background*. Low back pain (LBP) is a major cause of disability and the most common work-related musculoskeletal disorder among physiotherapists. This study examined the prevalence of low back pain among students undergoing training to become physiotherapists.* Methods*. Participants were 207 undergraduate clinical physiotherapy students at three universities in Nigeria. A modified version of a questionnaire used in a previous study was utilized to obtain demographic, educational activities, and LBP data. Prevalence of LBP was examined with descriptive statistics while factors associated with prevalence were explored using chi-square statistics.* Results*. More male students (53.1%) and those in the penultimate year of study (53.1%) participated in the study. Lifetime, 12-month, 1-month, and 7-day prevalence of LBP were 45.5%, 32.5%, 17.7%, and 11.5%, respectively. Prevalence of LBP was not significantly associated with any of the demographic variables. Educational activities, namely, “having techniques practiced on self for ≤10 hours” and “treating patients for ≥30 hours,” a month prior to the study were significantly (*P* < 0.05) associated with higher 1-month and 7-day LBP prevalence, respectively.* Conclusions*. Although the prevalence of LBP was comparatively low, its association with educational activities emphasizes the need to incorporate effective LBP preventive strategies in the training of physiotherapy students.

## 1. Introduction

Low back pain (LBP) is as old as humanity itself [1]. Over the years, the prevalence of the condition has been reported among different populations with particular interest in various occupational groups. From as early as the 17th century to date, LBP resulting from occupational and work-related activities has generated lots of attention in health care literature [1, 2]. It is however interesting to note that health care professionals are not exempt from the scourge of LBP. Consequently, several studies have focused on the prevalence of LBP among various health care professionals including physiotherapists [3–6].

Physiotherapists are professionals trained to, among other services, provide rehabilitative care in a wide range of disabling conditions with the aim of restoring, maintaining, and promoting function [7]. Interventions utilized by physiotherapists often entail a considerable amount of  “hands-on” techniques that are characterized by repetitive movements, prolonged standing, and somewhat difficult postures [3, 8]. Transferring and lifting patients are also common work activities in physiotherapy and such activities are considered risk factors for LBP and have been linked to its onset [3].

It is important to note that exposure to many of these physiotherapy work activities commences from the period of undergraduate physiotherapy training thus making LBP a likely occurrence among physiotherapy students. The potentially deleterious postures physiotherapy students assume during other training-related activities such as prolonged sitting during lectures or personal study and practical classes involving practice of tests and therapeutic techniques may also increase the risk of LBP [9].

With the potentially high risk of LBP among physiotherapy students, several studies have explored the subject matter in different parts of the world [8–10]. However in Nigeria, the most populous country on the African continent and the first country to have an undergraduate physiotherapy degree programme in the West Africa subregion, there appears to be little or no reporting on LBP among physiotherapy students. This study therefore examined the prevalence of LBP among undergraduate physiotherapy students in Nigeria. Potential associations between the prevalence of LBP based on the students' gender, age, and year of study and selected educational activities were also explored.

## 2. Materials and Methods

A cross-sectional design was used to survey physiotherapy students at three universities in Nigeria. The three universities were purposively selected from the seven universities in Nigeria with undergraduate physiotherapy programmes at the time of the study. The choice of the three universities was based on their location; one university was selected from each of the three regions of Nigeria (Western, Eastern, and Northern Regions) where the seven universities are located. Students were recruited into the study if they were in the clinical phase of their programme (a phase which comprises the 4th and 5th year of study and entails clinical placements and exposure to clinical practice) and if they expressed willingness to participate in the study.

### 2.1. Instrument

We utilized a self-administered questionnaire that contained extracts from the questionnaire utilized in the study by Nyland and Grimmer [9]. Data on age, gender, and year of study were obtained in the first section of the questionnaire. The second section of the questionnaire contained questions on the amount of time the students spent on specific educational activities within one month prior to the study. The activities were “sitting at lectures,” “sitting during personal study,” “practicing techniques on others,” “having others practice techniques on self,” and “treating patients.” The third section of the questionnaire enquired about 7-day, 1-month, 12-month, and lifetime prevalence of LBP with questions such as “have you had low back trouble at any time during 7 days?,” “have you had low back trouble at any time during the last month?,” “have you had low back trouble at any time during the last 12 months?,” and “have you ever had low back trouble?,” respectively.

### 2.2. Procedure

The study was approved by the relevant Research and Ethical Committee. Eligible students were approached during their free hours within their respective university premises by the first and second authors and a research assistant, each handling one university. Questionnaires were distributed and retrieved in bulk in order to ensure anonymity. Data collection took place from April to July 2010.

### 2.3. Statistical Analyses

Descriptive statistics of mean, standard deviation, frequencies, and percentages were utilized in presenting the students' demographic and LBP data. Pearson's chi-square statistics was utilized to examine associations between LBP prevalence and students' demographic characteristics and time spent engaging in educational activities at an alpha level of 0.05. Since the questions on educational activities were in the context of engagement in the activities within 1 month prior to the study, we examined associations between the educational activities and 1-month and 7-day prevalence of LBP only. For the purpose of the inferential statistics (Pearson's chi-square), the time spent on each educational activity in the questionnaire was dichotomized based on an estimation of the average time likely to be spent by the students on each activity in a month. Time (in hours) spent “sitting at lectures,” “practicing techniques on others,” “having others practice techniques on self,” and “treating patients” was dichotomized based on a subjective estimation of the time allotted for such activities in physiotherapy curriculum in Nigeria, while time for “sitting during personal study” was dichotomized based on observations made among physiotherapy students.

## 3. Results

207 out of the 290 eligible undergraduate clinical physiotherapy students participated in the study, giving a response rate of 71%. The mean age of the students was 25.35 ± 3.25 years and more students were males (53.1%) and in the 4th year of study (53.1%).  Table 1 shows details of the respondents' demographic characteristics.

Prevalence of LBP varied across the time points examined with lifetime, 12-month, 1-month, and 7-day prevalence being 45.5%, 32.5%, 17.7%, and 11.5%, respectively. None of demographic factors assessed was associated with the prevalence of LBP. However among the educational activities the students engaged in, only treating patients (*P* < 0.05) and having techniques practiced on self (*P* < 0.01) were significantly associated with 7-day and 1-month LBP prevalence, respectively (Table 2).

## 4. Discussion

The risk of physiotherapy students developing LBP may be similar to what obtains among physiotherapy practitioners, hence the need for adequate attention to their LBP status in research literature and to achieve LBP prevention as much as is possible.

Prevalence of LBP among physiotherapy students in this study was lower than that reported in existing studies from different parts of the world [8–10]. This comparatively lower prevalence notwithstanding, the discomfort and functional limitations that often accompany LBP should provide the required impetus for concerted efforts and strategies capable of preventing LBP. Planning and providing such preventive strategies may however depend, to a large extent, on identification of factors that significantly impact on the onset and prevalence of LBP.

Treating patients and having practical techniques practiced on self were the two educational activities that were significantly associated with 7-day and 1-month prevalence of LBP, respectively. While treating patients for 30 or more hours in the previous month was associated with increased prevalence of LBP, the converse was observed for the activity “having techniques practiced on self.” Physiotherapy interventions often entail repetitive movements, assuming awkward postures or working in static posture for prolonged periods, and manual handling, all of which have been implicated in the incidence and prevalence of LBP among physiotherapists [3, 4]. Our finding therefore appears to be in line with the submission that physiotherapy students are exposed to similar LBP risk factors as physiotherapy practitioners [4], though possibly at a lower frequency.

Unlike the explicable significant association between LBP prevalence and treating patients, the reason for the comparatively higher prevalence of LBP among students who had techniques practiced on them for shorter periods is unclear and may require further analysis in future studies. However, the fact that the prevalence of LBP among physiotherapy students in our study was associated with potentially modifiable factors implies the possibility of LBP prevention among the students.

Although posture is often implicated in the etiology of mechanical LBP, our study found no significant association between prevalence of LBP and sitting for lectures and personal studies. Our study however only focused on length of time spent sitting and not on the appropriateness or otherwise the sitting posture adopted. It is important to state that while LBP has been linked to awkward sitting posture, there appears to be insufficient research evidence on the association between length of sitting and LBP [2]. Future studies may consider investigating the types of sitting postures assumed by physiotherapy students and the impact of such postures on LBP occurrence and prevalence.

Our findings showed no significant association between prevalence of LBP and nonmodifiable factors such as the students' age and gender. Previous reports on the influence of demographic variables on prevalence of LBP among physiotherapy students are however conflicting [8, 9].

### 4.1. Limitations of the Study

The nonprobability sampling technique used in recruiting the respondents in this study may have resulted in selection bias and may limit generalizability of our findings. The small size of our sample also precluded the use of more rigorous statistics such as regression analysis to identify independent factors that influenced LBP prevalence.

## 5. Conclusion

The prevalence of LBP in our study, coupled with the educational activities found to be associated with the prevalence, indicates that issues of effective LBP prevention strategies should be addressed in undergraduate physiotherapy education and training.

## Figures and Tables

**Table 1:** Demographic characteristics of respondents (*N* = 207).

Variable	Value
Age (years)	
Mean ± SD	25.35 ± 3.25
Age range	20–47

	*f* (%)

Gender	
Male	110 (53.1)
Female	97 (46.9)
Year of study	
4th	110 (53.1)
5th	97 (46.9)

**Table 2:** Associations between prevalence of LBP and ^*∗*^exposure to specific educational activities.

Activity	Low back pain prevalence	
	7-day prevalence (%)	1-month prevalence (%)
Sitting (lectures)		
<50 hours	11.2	16.9
≥50 hours	14.8	25.9
*χ* ^2^	**0.29**	**1.30**
*P* value	**0.59**	**0.25**
Sitting (personal study)		
<50 hours	11.5	18.8
≥50 hours	18.2	1.9
*χ* ^2^	**0.44**	**0.66**
*P* value	**0.51**	**0.42**
Practicing techniques on others^a^		
≤10 hours	6.1	19.5
>10 hours	13.4	15.2
*χ* ^2^	**4.20**	**0.80**
*P* value	**0.12**	**0.67**
Others practicing techniques on self^b^		
≤10 hours	11.9	20.2
>10 hours	10.3	5.9
*χ* ^2^	**0.15**	**11.10**
*P* value	**0.93**	**0.004** ^##^
Treating patients^c^		
<30 hours	6.3	19.8
≥30 hours	20.3	16.5
*χ* ^2^	**9.21**	**0.71**
*P* value	**0.01** ^#^	**0.70**

^*∗*^Exposure to educational activities in the previous month.

^a,b,c^Not all the respondents indicated exposure to these activities; for those that did we have the following: a = 149 respondents; b = 141 respondents; c = 190 respondents.

^#^Statistically significant at *P* < 0.05; ^##^statistically significant at *P* < 0.01.
